# Benchmarking and optimizing qualitative and quantitative pipelines in environmental metatranscriptomics using mixture controlling experiments

**DOI:** 10.1093/ismeco/ycaf090

**Published:** 2025-05-29

**Authors:** Weiyi Li, Qilian Fan, Yi Yang, Xiang Xiao, Jing Li, Yu Zhang

**Affiliations:** School of Life Sciences and Biotechnology, Shanghai Jiao Tong University, Shanghai 200240, China; School of Life Sciences and Biotechnology, Shanghai Jiao Tong University, Shanghai 200240, China; School of Life Sciences and Biotechnology, Shanghai Jiao Tong University, Shanghai 200240, China; School of Life Sciences and Biotechnology, Shanghai Jiao Tong University, Shanghai 200240, China; School of Life Sciences and Biotechnology, Shanghai Jiao Tong University, Shanghai 200240, China; State Key Laboratory of Submarine Geoscience; Key Laboratory of Polar Ecosystem and Climate Change, Ministry of Education; Shanghai Key Laboratory of Polar Life and Environment Sciences; and School of Oceanography, Shanghai Jiao Tong University, 1954 Huashan Road, Shanghai 200030, China; SJTU Yazhou Bay Institute of Deepsea Sci-Tech, Shanghai Jiao Tong University, Sanya, Hainan 572100, China

**Keywords:** metatranscriptomic analysis, mock communities, environmental microbiome, pipeline, optimization, quantitative analysis, taxonomic profiling

## Abstract

Metatranscriptomic analysis is increasingly performed in environments to provide dynamic gene expression information on ecosystems, responding to their changing conditions. Many computational methods have undergone remarkable development in the past years, but a comprehensive benchmark study is still lacking. There are concerns regarding the accuracies of the qualitative and quantitative profilers obtained from metatranscriptomic analysis, especially for the microbiota in extreme environments, most of them are unculturable and lack well-annotated reference genomes. Here, we presented a benchmark experiment that included 10 single-species and their cell or RNA-admixtures with the predefined species compositions and varying evenness, simulating the low annotation rate and high heterogeneity. In total, 1 metagenome sample and 24 metatranscriptome were sequenced for the comparisons of 36 combination of analysis methods for tasks ranging from sample preparation, quality control, rRNA removal, alignment strategies, taxonomic profiling, and transcript quantification. For each part of the workflow mentioned above, corresponding metrics have been established to serve as standards for assessment and comparison. Evaluation revealed the performances and proposed an optimized pipeline named MT-Enviro (MetaTranscriptomic analysis for ENVIROnmental microbiome). Our data and analysis provide a comprehensive framework for benchmarking computational methods with metatranscriptomic analysis. MT-Enviro is implemented in Nextflow and is freely available from https://github.com/Li-Lab-SJTU/MT-Enviro.

## Graphical Abstract

**Graphical Abstract:**
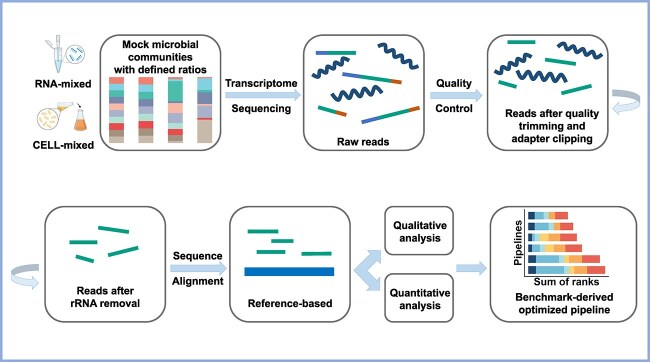

## Introduction

The uncharted microbial world extends beyond the deep seas to myriad diverse environments across Earth. These environments span various extremes, and microorganisms from these diverse ecosystems are of significant interest due to their resilience to different conditions and potential for various applications [1, 2]. Moreover, microorganisms play a crucial role in ecosystem maintenance and elemental cycling. Their ability to adapt to extreme and fluctuating conditions makes them a rich source of novel biochemical pathways [3]. However, many of these microorganisms are challenging to isolate and cultivate in the laboratory, thereby increasing academic interest in the meta-omics analysis of these diverse microbiomes [4, 5]. While metagenomic analysis provides insights into the functional potential of microorganisms [6], metatranscriptomic analysis reveals the active genes within a microbial community [7].

Nonetheless, challenges persist in the meta-omics analysis of these complex and diverse microbial communities. Factors such as low annotation rates, high heterogeneity, and the absence of defined references with known microbial communities limit the accuracy and reliability of these analyses [1, 2, 8–10]. Therefore, it is crucial to design a dataset that simulates the characteristics of real environmental samples to serve as a benchmark for qualitative and quantitative evaluation. *In silico* simulated datasets, constructed by randomly selecting genes from combined transcriptome databases, have been used to evaluate bioinformatics analysis tools [11, 12]. However, these *in silico* simulated datasets do not include information on laboratory and biological effects. Datasets derived from real biological samples often lack the necessary information for quantitative comparison [13]. Mock microbial communities—artificially assembled mixtures of known microorganisms with defined RNA abundance ratios—offer a promising middle ground by simulating real experimental conditions while providing ground truth for both community composition and quantitative evaluation.

**Figure 1:**
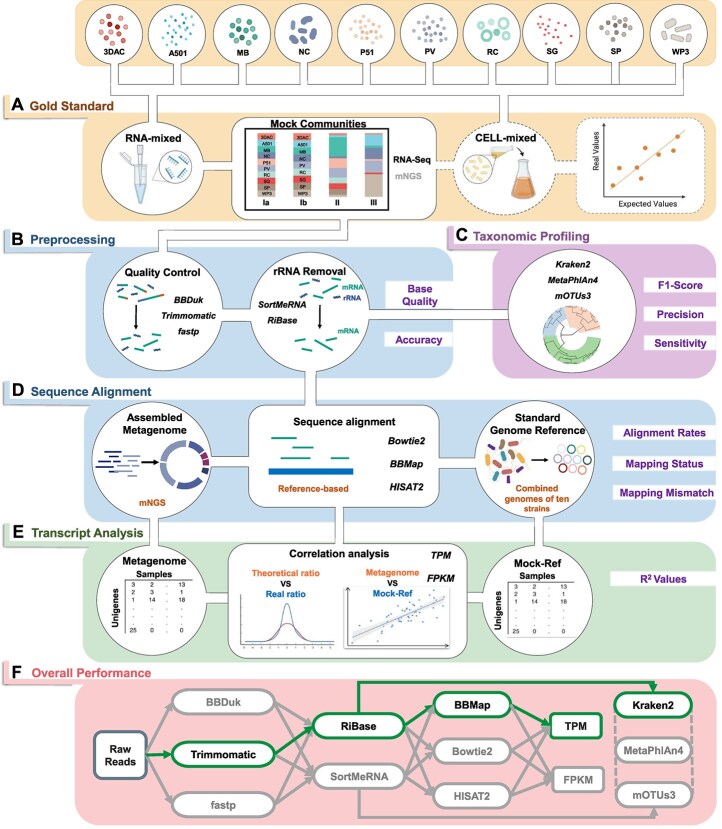
**Comprehensive flow chart of MT-Enviro.** Italic text denotes the tools used in this step, while purple text indicates the metrics to evaluate the step. **A** Mock microbial communities are constructed from ten known strains. Samples are obtained from different mixing methods and proportions. Metatranscriptomic sequencing was performed for three replicates of each group. **B** Preprocessing of the reads consisting of two steps: quality control and rRNA removal. **C** Taxonomic profiling was performed by three profilers with reads after preprocessing. **D** The reference genome was derived from the metagenome obtained by the direct sequencing of the bacterial mixture (Assembled Metagenome) or the combined information of the individual genomes of ten known bacterial species (Standard Genome Reference). **E** Transcriptomic quantification is represented by normalized values such as TPM/FPKM, and a comparison is made between theoretical and empirical values. **F** Considering the overall performance across all pipelines, the recommended pipeline is depicted by green lines.

In this study, we built a set of mock microbial communities with predefined species compositions and varying evenness. This community dataset was designed to replicate the low annotation rate and high heterogeneity characteristic of microbiomes from diverse environments (Fig. 1). The goal is to use this dataset, which simulates the characteristics of real-world samples, as a benchmark to evaluate the accuracy of species annotation and gene quantification in existing analysis pipelines. The mock communities were prepared using two methods: by mixing cells directly before RNA extraction (cell-mixed) and by mixing RNA extracted from individual strains (RNA-mixed). Utilizing the benchmarking datasets we evaluated the metatranscriptomic analysis processes, including sample preparation, quality control, rRNA removal, alignment strategies, taxonomic profiling, and transcript quantification. Ultimately, we proposed an optimized analysis process, named “MT-Enviro”, which is suitable for most common analysis steps for metatranscriptomic data derived from a diverse range of environments.

## Materials and methods

### Strains and cultivation

Ten species that are primarily distributed across marine, soil, and extreme environments were selected (Table 1). Each strain was cultured in 100 ml solution as a culture unit. The OD_600_ of one of the bottles was measured to document and plot the growth curve as a control.

**Table 1:** Composition of mock microbial communities.

Strain	Strain Abbreviation	Kingdom	Cell type	Genome Size (Mb)	Distribution	RNA mass (1 = 1 μg RNA)			
						I. evenly		II. High	III. Low
						a.10 strains	b.9 strains	evenness	evenness
***Zhurongbacter thermophilus* 3DAC**	3DAC	Bacteria	G-	1.6	Deep-sea hydrothermal vent	1	1	1	5
***Thermococcus eurythermalis* A501**	A501	Archaea	G-	2.1	Deep-sea hydrothermal vent	1	1	1	50
***Marinobacter hydrocarbonoclasticus* MT11–1**	MB	Bacteria	G-	4.0	Marine environment	1	1	10	10
***Haloferax volcanii* DS2**	NC	Archaea	G+	3.9	High-salinity aquatic environments	1	1	1	50
***Pseudomonas gessardii* 5–1**	P51	Bacteria	G-	6.6	Marine, natural mineral waters, soil, environmental pollutants, etc.	1	0	5	8
***Pseudomonas veronii* 2–3**	PV	Bacteria	G-	7.3	Marine, soil, environmental pollutants, etc.	1	1	5	50
***Rhodococcus sp.* MT11–4**	RC	Bacteria	G+	5.2	Marine, soil	1	1	3	1
***Sanguibacter keddieii* 11–6**	SG	Bacteria	G+	4.1	Marine, soil	1	1	3	8
***Sphingomonas profundi* LMO-1**	SP	Bacteria	G-	4.5	Marine sediment	1	1	3	1
***Shewanella piezotolerans* WP3**	WP3	Bacteria	G-	5.3	Marine environment	1	1	1	100

G− Gram Negative

G+ Gram Positive

Cells were harvested in the mid-log growth phase by centrifugation (10 min at 12 000 rpm, 4°C). Before centrifugation, all of the culture units for the same species were mixed in a 2 L conical flask and shaken well before dispensing into 50 ml centrifuge tubes (DNase & RNase free) and quickly placed in prechilled −70°C ethanol. After centrifugation, the supernatant was discarded, and the cells were kept at −80°C (Supplementary Fig. S1).

### RNA extraction

Total RNA from the collected microbial cells was extracted using a modified TRIzol protocol (Invitrogen, Carlsbad, CA, USA). Briefly, the collected cells (50 ml as a unit) were resuspended in 1 ml of TRIzol and transferred to a prechilled 2 ml microcentrifuge tube with 0.2 g glass beads. Then, the tube was placed in the FastPrep-24 homogenizer (MP biomedicals, Irvine, CA, USA), and the cells were disrupted using the beads and rotor. After disruption, the RNA was purified using trichloromethane, isoamyl alcohol, and 75% ethanol. The purified RNA was dissolved in 80 μl DEPC (diethyl pyrocarbonate) water and further treated with DNase I (Qiagen, Hilden, Germany) for 1 hour at 37°C to break down residual DNA, followed by purification with 75% ethanol again. The final product was redissolved in 150 μl DEPC water, and RNA samples from the same strain were combined in one tube. The concentration of RNA was quantified by a Qubit RNA HS Assay Kit with a Qubit 4.0 fluorometer (Invitrogen, Carlsbad, CA, USA), and the quality was checked by gel electrophoresis.

### Formulation of the RNA-mixed mock community

Extracted RNA from each strain was combined to obtain RNA mock communities with different evenness (Table 1) and stored at −80°C until use. Each mock community was combined three times in volume and then divided into three equal parts after mixing. All procedures were performed on ice at low temperature and under RNase-free conditions. Finally, the RNA concentration of each RNA-mixed mock community was measured using Qubit 4.0 (Supplementary Table S1).

### Formulation of the cell-mixed mock community

The harvested cells were thawed from −80°C at room temperature and resuspended in 50 ml of PBS (phosphate buffer saline) solution per tube (50 ml as a unit). The theoretical volume of liquid culture for each strain in the cell-mixed mock communities was estimated based on the theoretical RNA addition amounts and the experimentally determined RNA extraction efficiency for each strain at a given OD_600_ (Supplementary Table S1). Then, the mixed samples were centrifuged at 12 000 rpm for 15 min at 4°C, the supernatant was discarded, and total RNA was extracted. Finally, the RNA concentration of cell-mixed samples was measured using a Qubit 4.0 instrument. The extracted RNA was snap frozen using liquid nitrogen and stored at −80°C until use.

### Library construction and sequencing

rRNA depletion was performed using the ALFA-SEQ rRNA Depletion Kit (for Bacteria), developed by MAGIGENE (Guangzhou, China), which is based on the Illumina Ribo-Zero Plus rRNA Depletion Kit and targets bacterial 5S, 16S, and 23S rRNA. Library preparation was conducted using the NEBNext® Ultra II Directional RNA Library Prep Kit for Illumina® (New England Biolabs, Ipswich, MA, USA), followed by sequencing on the Illumina HiSeq 2500 platform (Illumina, San Diego, CA, USA). All procedures, including rRNA depletion, library construction, and sequencing, were performed by MAGIGENE (Guangzhou, China), generating 150 bp paired-end reads.

The rRNA transcripts were eliminated by ALFA-SEQ rRNA depletion Kits (for Bacteria) for depleting Bacteria 5S, 16S, and 23S rRNA. Ribosomal RNA depletion with the Ribo-Zero™ rRNA Removal Kit (Epicentre, Madison, WI, USA), library preparation with the NEBNext® Ultra IITM Directional RNA Library Prep Kit for Illumina® (New England Biolabs, Ipswich, MA, USA), and sequencing on the Illumina HiSeq 2500 platform (Illumina, San Diego, CA, USA) were all performed by Magigene Company (Guangzhou, China), and 150 bp paired-end reads were generated.

### Reference database from individual genomes of defined species

For each of the selected species, a high-quality reference genome was available. These reference genomes were merged to create the defined reference database, Mock-Ref, for these environmental microbial communities. The merged genome file (mix.fasta, FASTA format) was used to generate the protein-coding DNA sequence file (mix.fna, FASTA format), gene annotation file (mix.gtf, GTF format), and protein sequence file (mix.faa, FASTA format) using Prodigal (v2.7.1, RRID:SCR_011936) [14].

Redundancy was removed from the protein sequence file (mix.faa, FASTA format) using Cd-hit (v4.8.1, RRID:SCR_007105) [15]. In the subsequent transcripts per million (TPM) calculation, for conserved genes present across multiple strains, we were unable to distinguish the contribution of each strain to the TPM value of these genes. Therefore, to facilitate the subsequent quantitative analysis using read alignment results and to compare the relative abundance of the same species across different samples, we need to obtain information on the unique genes of each species and use the TPM values of these unique genes to represent the quantitative performance of the species. The clustering threshold of Cd-hit was set as 0.4 to obtain the unique gene sequences (unigene.fasta, FASTA format) of each species in the mock communities.

### Metagenome reference database

The harvested cells were thawed at room temperature from −80°C and resuspended in 50 ml PBS per tube. Equal volumes of cell cultures from each strain were mixed to generate the mock community for metagenomic analysis. The mixture was centrifuged at 12 000 rpm for 15 min at 4°C, and the supernatant was discarded. Total DNA was extracted using either the PowerSoil DNA Isolation Kit (Qiagen, Germantown, USA) or the FastDNA® Spin Kit for Soil (MPBIO, USA), according to the manufacturers’ protocols. DNA was snap frozen in liquid nitrogen and stored at −80°C until use.

Metagenomic libraries were prepared using the TruSeq Nano DNA LT Library Prep Kit (Illumina, USA) with index barcoding, following the manufacturer’s instructions. Paired-end sequencing (150 bp) was performed on the Illumina NovaSeq 6000 platform (Illumina, USA) by MAGIGENE (Guangzhou, China).

Metagenomic raw reads were trimmed using BBDuk. Filtered-read-quality was estimated by FastQC (v0.11.9) [16]. Metagenomic contigs were assembled from clean reads using Megahit (v1.2.9, RRID:SCR_018551) [17]. The assembled metagenome was compared with the pooled standard genomes by MUMmer [18]. Protein-coding DNA sequences were determined using Prodigal (v2.7.1) [14], and a reference database was constructed from the metagenome (MG_genome.fasta), as was a gene annotation file (MetaGenome.gtf) and protein sequence file (MetaGenome.faa).

### Construction of a non-redundant rRNA database

Ribosomal RNA sequence databases were used for RiBase, including Greengenes2 (2024.9) [19], RDP (v11.5, 2016.9.10) [20], Rfam (v15.0, 2024.11) [21], SILVA (v138.2, 2024.7) [22, 23], and NCBI 16S rRNA sequences (PRJNA33317, PRJNA33175). All sequences were downloaded and merged into a uniform format using riboPicker scripts [24]. The redundant rRNA sequences were trimmed using prinseq-lite.pl from PRINSEQ [25] after the sequence files were merged. The default database of SortMeRNA, smr_v4.3_default_db.fasta, was used for the comparison of rRNA removal results [26].

### Evaluation of rRNA removal efficiency

To evaluate the performance of rRNA removal methods, we utilized the gold-standard rRNA database constructed from the rRNA sequences of the 10 strains used in this study. For rRNA identified by RiBase and SortMeRNA, comparisons were made against this gold-standard database:

Sequences identified by the tools and present in the gold-standard rRNA database were considered true positives (TP). rRNA sequences present in the gold-standard database but not identified by rRNA removal methods were considered false negatives (FN), indicating that the method failed to detect all rRNAs. Sequences identified by the methods but absent from the gold-standard rRNA database were considered false positives (FP), meaning that the method falsely classified some mRNA reads as rRNA.

Alternatively, we aligned the sample reads against the gold-standard rRNA database constructed from the 10 strains to identify the rRNA reads in each sample. Precision is the proportion of correctly identified rRNA reads against total reads (TP / TP + FP). Sensitivity signifies the proportion of the real rRNA dataset effectively covered by the rRNAs identified by the two approaches (TP / TP + FN).

### Metatranscriptomic data analysis

Sequencing saturation curves: 10%, 20%, 30%, to 100% of the reads were randomly selected using Picard’s DownsampleSam tool (v2.27.5, https://broadinstitute.github.io/picard/). For each set of samples, the total number of aligned genes was counted separately using featureCounts (RRID:SCR_012919) from the Subread package (v2.0.1, RRID:SCR_009803) [27].

Data preprocessing: Raw reads were trimmed for quality, read length, and adapter content by Trimmomatic (v0.39) [28], BBDuk (from the BBTools suite v38.87), and fastp (v0.21.0) [29], respectively, with average quality scores ≤20 and lengths ≤50 bp. The quality of clean reads was reported by FastQC and MultiQC (v1.11) [30]. Each FastQC section includes a status showing whether the results seem entirely normal, slightly abnormal or very unusual. The results of the FastQC sections “Basic Statics”, “Per Tile Sequence Quality”, “Per Base Sequence Content”, “Per Sequence GC Content”, “Sequence Duplication Levels”, and “Overrepresented sequences” were counted as the Base Quality group. The results of the FastQC section “Adapter Content” were counted as the Adapter Removal. Ribosomal RNA sequences were removed by alignment to RiBase using Bowtie2 (v2.4.2) [31]. SortMeRNA (version 1.2) [26] was also used to filter out rRNA sequences using the default databases for comparison.

Reference-based alignment: HISAT2 (v2.2.1, RRID:SCR_015530) [32], BBMap (from the BBTools suite v38.87, RRID:SCR_016965), and Bowtie2 (v2.4.2, RRID:SCR_016368) [31] were used to map reads to the reference database constructed from the 10-species genomes or from the metagenomes. Reference coverage and average read depth were extracted using SAMtools (v1.14, RRID:SCR_002105) [33]. Then, featureCounts [27] was used to count the reads. With parameters maintained at default values, featureCounts does not count reads overlapping with more than one feature. Raw counts were further normalized to TPM values [34].

The list of tools, their versions, and the command line options used in the metatranscriptomic analysis are recorded in Supplementary Table S2. All MT-Enviro scripts were run on a high-performance computing system built with Intel Cascade Lake CPUs. Configuration parameters: CPU: 2 x Intel Xeon Cascade Lake 6248 (2.5 GHz, 20 cores); Mem: 12 x Samsung 16GB DDR4 ECC REG 2666.

### Taxonomic classification

Following rRNA depletion, high-quality sequencing reads were processed through three taxonomic classifiers: Kraken2 [35] utilizing the RefSeq non-redundant protein database (release 09/2018, 30 GB), MetaPhlAn4 [36] with the mpa_vJun23_CHOCOPhlAnSGB_202403 database and mOTUs3 [37] employing the db_mOTU_v3.1.0 database.

### TPM ratio linear fit

In section “Transcript quantification”, for the analysis using the pooled reference genomes composed of 10 selected strains (Mock-Ref) as a reference, the TPM values of the same unique gene group by strain were compared between samples (Ib vs. Ia; II vs. Ia; III vs. Ia) to obtain the TPM ratio. During the construction of the RNA-mixed samples, we strictly followed the proportions specified in Table 1 for the RNA of each species. The descriptions of samples and their corresponding abbreviations are recorded in Supplementary Table S1. The proportion of each species in the sample was determined based on the amount of RNA added for each species relative to the total RNA content in the sample. For example, the Expected RNA proportion of *Thermococcus eurythermalis* A501 in sample Ia is 1/10, while in sample Ib it is 1/9 (Table 1).

Furthermore, we compared the relative abundance of each species across different samples. For each strain, under the condition where only the amount of RNA added varies, with all other laboratory conditions held constant, we assumed that Expected TPM Ratio for each species’ unique genes between different samples reflects the ratio of RNA abundance for that species in the samples. The Expected TPM Ratio is calculated based on the planned mixing ratios of RNA from different strains, as specified in Table 1, representing the theoretical expected values under ideal conditions. For instance, the expected TPM ratio of *Thermococcus eurythermalis* A501 between Ib and Ia samples is $\frac{1}{9}\div \frac{1}{10}\approx 1.11$. We then grouped the species and calculated the quantitative differences in the TPM values of each species’ unique genes across different sample pairs as the Real TPM Ratio. The values span several orders of magnitude, thus we decided to fit the monomial functional for easier fitting as a linear model:


$$\begin{array}{l} {\log}_2\left(\mathrm{Real}\ \mathrm{TPM}\ \mathrm{Ratio}+0.0001\right)\\=\mathrm{k}\bullet{\log}_2\left(\mathrm{Expected}\ \mathrm{TPM}\ \mathrm{Ratio}+0.0001\right)+b \end{array}$$

### TPM linear fit

To compare the TPM values from different references (Mock-Ref, the metagenome and Uniref90), the linear model was designed as follows:


$$ \mathrm{TPM}\ \mathrm{from}\ \mathrm{metagenome}=k\bullet \left(\mathrm{TPM}\ \mathrm{from}\ \mathrm{Mock}\ \mathrm{Ref}\right)+b $$

$$ \mathrm{TPM}\ \mathrm{from}\ \mathrm{Uniref}90=k\bullet \left(\mathrm{TPM}\ \mathrm{from}\ \mathrm{Mock}\ \mathrm{Ref}\right)+b $$

The release version of the UniRef90 database used in this study is: 2024_06, 27-Nov-2024, Number of clusters: 199553294 [38]. For the same unique gene, the TPM value obtained from the metagenome or Uniref90 was used as y, and the TPM value obtained from the pooled standard genome was used as x in the linear model.

### Evaluation metrics

To evaluate taxonomic assignment, we computed the F1 score, Precision, and Sensitivity for each profiler. Leveraging the metrics and scripts provided in the original Kraken2 publication [36, 40], we assessed taxonomic classification accuracy on a per-fragment basis across specific taxonomic ranks. The Kraken2 framework supports evaluating classification performance from the phylum to genus levels; in our analysis, we focused on the genus level. Classification outcomes were categorized as follows:

(i) TP: The assigned taxon matches the true genus of origin or one of its descendant taxa; (ii) FN: The classifier failed to assign any taxonomic label to the fragment; (iii) VP: The assigned classification corresponds to an ancestor of the true genus; (iv) FP: The classification is incorrect, i.e. it does not correspond to the true genus or any of its ancestors or descendants. Following Kraken2’s taxonomy-aware framework, we defined rank-level sensitivity as TP / (TP + VP + FN + FP), precision (PPV) as TP / (TP + FP), and F1-score as the harmonic mean of sensitivity and precision.

For MetaPhlAn4 and mOTUs3, we conducted comparative analyses with Kraken2 by evaluating species identification accuracy through precision, recall, and F1-score metrics. Given that MetaPhlAn4 and mOTUs3 rely on marker gene reference databases rather than comprehensive genomic libraries, the standard Kraken2 evaluation framework above was not directly applicable. To address the intrinsic characteristics of taxonomic profilers’ annotation outputs, precision was specifically defined as the proportion of true-positive species relative to the total species identified by each profiler. To calculate the sensitivity, we independently processed the MetaPhlAn4 and mOTUs3 databases through a two-stage refinement protocol. For each analytical tool, all marker genes were first aligned to the Mock-Ref genome using stringent criteria (E-value <1e-5; ≥97% coverage of each marker gene’s full length). Subsequently, we identified reads classified by either MetaPhlAn4 or mOTUs3 that aligned to their corresponding Mock-Ref marker gene set in the complete Mock-Ref reference alignment. Sensitivity was calculated as the ratio of reads both identified by each taxonomic profiler and aligned to the Mock-Ref marker gene set, relative to all marker-aligned reads in the respective database.

## Results

### Defined mock communities enable qualitative and quantitative evaluation

**Figure 2:**
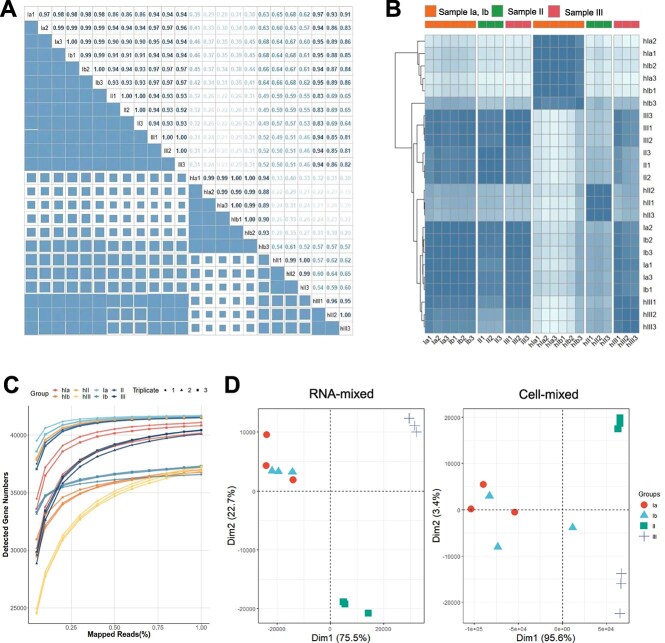
**Evaluation analyses of defined mock communities. A** Pearson correlation coefficient plot. **B** Heatmap of gene expression correlations. The colour in the heatmap represents the correlation coefficient. Hierarchical clustering based on quantitative gene expression results among samples was performed on the rows of the heatmap. Columns are separated according to the mixed-sample approach and homogeneity. **C** Sequencing saturation curve of defined mock communities. **D** PCA plots of 24 metatranscriptomics samples. Samples from different mixing methods are shown separately. Triplicates of each group are plotted as points of the same shape.

For the establishment of mock communities, we selected 10 species that are distributed across marine, soil, and extreme environments (Table 1). These strains were mixed in varying proportions to assess the effect of species abundance on taxonomy and alignment. Two sample mixing methods were implemented at four evenness levels (Materials and Methods). Twenty-four metatranscriptomes with a total of ~4.17 billion 150 bp paired-end reads were obtained. To measure the quality of the sequencing data, the consistency among sample triplicates was of primary interest. Pearson correlation analysis was performed for the correlation of TPM values in different samples (Fig. 2A). The triplicates were strongly correlated, with correlation coefficients ranging from 0.928 to 1 (*P* value <2.2e-16).

Hierarchical clustering of samples was performed according to the transcriptional quantitative information (Fig. 2B). Samples with the same mixing method and the same degree of homogeneity were clustered together. Similar results were obtained in the principal component analysis (PCA, Fig. 2D), where Ia and Ib samples were distributed close together and separated from the other two types of samples (II, III).

The sequencing saturation curves of the defined mock communities converged to straight lines as the sampling scale approached 100% (Fig. 2C). This indicated that additional sequencing could not identify more expressed genes, and the obtained results could be used to guarantee the accuracy of subsequent quantitative analysis.

In meta-omics studies, metagenomic data are often combined with metatranscriptomic data for taxonomic profiling, read alignment, and transcript quantification [7, 39]. To validate the real-world applicability of MT-Enviro, we simultaneously sequenced a metagenomic dataset while constructing the metatranscriptomic dataset. Metagenomic next-generation sequencing was performed on evenly cell-mixed sample Ia, and ~ 2.23 billion 150 bp paired-ended reads were obtained. For this metagenome, any two predicted genes with >95% identity or coverage of more than 90% of the shorter gene were clustered together [40], resulting in a set of 34 376 non-redundant genes. We further compared the assembled metagenome with the pooled reference genomes composed of 10 selected strains (Mock-Ref) to assess the quality of the metagenome using MUMmer (RRID:SCR_018171) [18]. In total, 99.68% of the metagenomic sequences could be aligned in Mock-Ref and accounted for 83.90% of this reference genome.

### Evaluation of RNA acquisition under two mixing modes

In metatranscriptomic studies, samples were obtained mainly from bacterial mixes [41]. However, the RNA acquisition efficiency, the RNA degradation and the quantitative accuracy of the RNA obtained from the mixed bacterial solution requires further validation. To achieve a more precise quantitative assessment, MT-Enviro employed two mixing methods: cell-mixing and RNA-mixing. By measuring the actual RNA amount in the mixed samples and comparing it with the theoretical value, the quantitative accuracy of the obtained RNA can be determined.

The theoretical RNA amount represents the expected total RNA in each sample, calculated based on the RNA contributed by each strain, as shown in Table 1. It serves as a reference for subsequent steps, while actual RNA amounts may vary due to extraction efficiency, handling, or transportation. The actual RNA amount after mixing is calculated by multiplying the RNA concentration by the sample volume, helping to assess RNA recovery and potential losses. After transportation, the measured total RNA helps evaluate sample stability and potential degradation.

The actual RNA amounts, including both RNA amount after mixing and RNA amount after transporting, along with the theoretical values of the mixed samples, are recorded in Supplementary Table S1, where Ia, Ib, II, and III represent the RNA-mixed samples, and hla, hIb, hII, and hIII represent the cell-mixed samples (Materials and Methods). The results showed a strong positive correlation between the theoretical and actual values of the RNA-mixed samples (Spearman’s rank correlation coefficient = 1, *P*-value <2.2e-16). For the cell-mixed samples, although a positive correlation was also observed (Spearman’s rank correlation coefficient = 0.972, *P*-value = 1.381e-07), it was difficult to determine the exact contribution of each strain, and there were significant discrepancies between the theoretical and extracted values due to differences in transportation and extraction processes. Therefore, to ensure the reliability of subsequent assessments, we used RNA-mixed samples as the raw data for evaluation.

### Performance of quality control tools

We used three quality control tools, Trimmomatic (RRID:SCR_011848) [28], BBDuk (from the BBTools suite v38.87, RRID:SCR_016969), and fastp (RRID:SCR_016962) [29], to remove the raw reads with low quality and adaptor contamination (Fig. 1B). MultiQC (RRID:SCR_014982) [30] was adopted to compare and assess the results before and after quality control in terms of adapter removal, base quality, running speed, etc.

**Figure 3:**
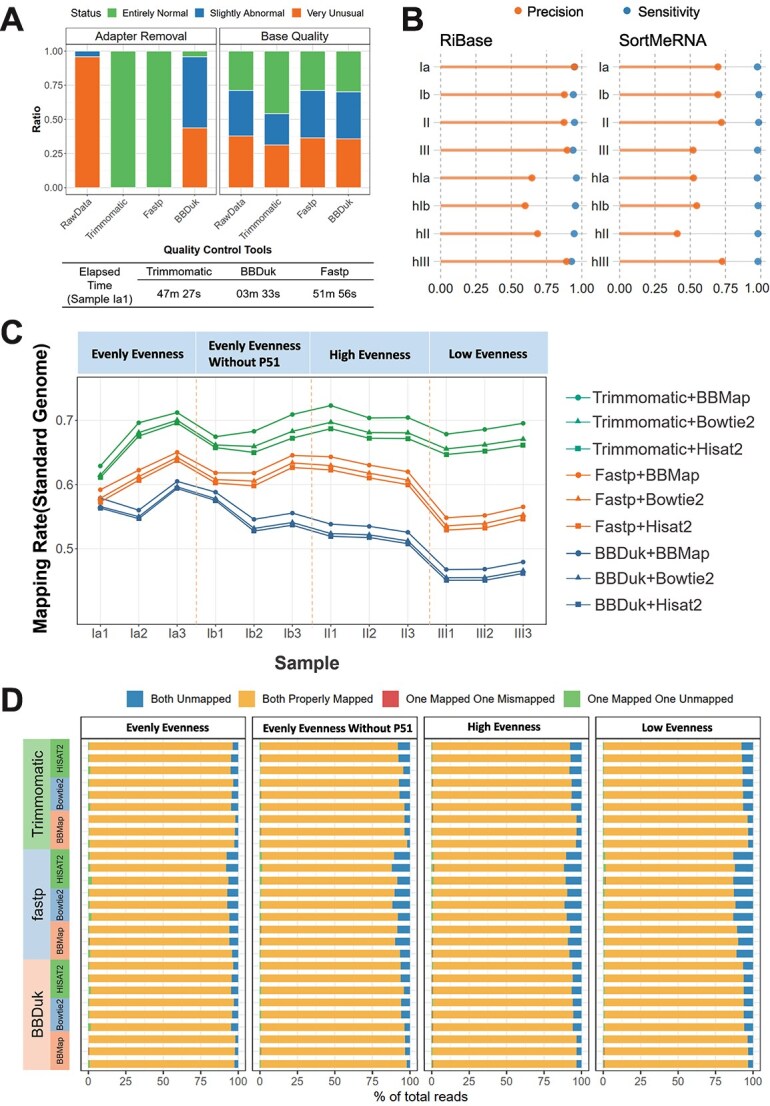
**Performance of different preprocessing and sequence alignment pipelines. A** Trimmomatic, BBDuk and fastp were used for quality control comparisons. Their improvements in base quality and adapter removal are shown in a bar plot. The speed of the tools is shown in the table. **B** Precision and sensitivity of different rRNA removal methods based on the standard rRNA reference. **C** Nine pipelines are used to obtain the mapping rate of reads by taking the standard genome as the reference genome. The results from each quality control pipeline are labelled with different colours, and the results from the alignment pipelines are labelled with different dot shapes. Different types of RNA mixing evenness are shown at the top and are divided by dashed lines. **D** Distribution of mapping status of nine reference-based alignment pipelines, labels on the left reflect the quality control tools (Trimmomatic, BBDuk and fastp), and the aligner used (BBMap, Bowtie2, HISAT2), different evenness of RNA mixing are shown at the top.

An average of 22.29% of raw reads was filtered in results from BBDuk. Trimmomatic filtered 8.85% of the total reads, while fastp filtered 1.63% (Supplementary Table S3). Furthermore, the comparison were based on the status of the read quality classified by FastQC (RRID:SCR_014583) [30] (Materials and Methods). We counted the samples with different quality statuses before and after quality control (Fig. 3A). All the samples were categorized as entirely normal after adapter removal with Trimmomatic or fastp, while only 8.33% entirely normal samples were observed with BBDuk. In terms of base quality, Trimmomatic performed the best by increasing the proportion of entirely normal samples from 28.82% to 45.83%. To directly compare the running time of the programs, we ran the same sample (Ia1) using all three tools. Fastp took the longest time, which was approximately 24 times longer than the time required by BBDuk (Fig. 3A).

### Enhancing rRNA removal efficiency with RiBase

Next-generation RNA sequencing (RNA-seq) analysis requires the enrichment of messenger RNA (mRNA) [42]. So effective rRNA removal is crucial for metatranscriptomic analysis. To improve rRNA removal efficiency, we established RiBase, an integrated rRNA dataset (Fig. 1B). RiBase contains rRNA sequences from public rRNA databases (Materials and Methods). To assess the reliability of rRNA removal by RiBase, our metatranscriptomes were also analysed using SortMeRNA (RRID:SCR_014402), a common tool employed for rRNA removal in previous metatranscriptomic studies [26, 43–45]. The analytical accuracy of both approaches was assessed using the rRNA dataset containing the full rRNA sequences of the 10 strains in the mock microbial community.

To assess reliability, we focused on two critical metrics: precision and sensitivity. Precision is the proportion of correctly identified rRNA reads among all reads classified as rRNA. Sensitivity signifies the proportion of the real rRNA dataset effectively covered by the rRNAs identified by the two approaches. (Fig. 3B, Materials and Methods). The precision of the RiBase results was higher than that of SortMeRNA (Wilcoxon signed rank test, *P* = 1.49e-06), with a mean increase of 0.198. Additionally, the sensitivity of the RiBase was slightly lower than that of SortMeRNA, with a mean difference of −0.035. However, the mean sensitivity of RiBase was 0.945, which was sufficient to indicate the completeness of the results.

### Evaluation and optimization of the reference-based alignment pipelines

To determine the best reference-based alignment pipeline, eighteen combinations of three quality control tools (Trimmomatic, BBDuk and fastp), two rRNA removal methods (RiBase and SortMeRNA), and three alignment tools (BBMap, Bowtie2 and HISAT2) were tested.

First, we counted the proportion of expressed genes of each bacterium separately and calculated the gene detection rate from read alignment results (Supplementary Table S4). The detection rates of most samples were high, indicating that each bacterium presented a high transcription rate. The choice of different alignment tools did not have a significant effect on the detection rate. However, the gene detection rates of *Pseudomonas gessardii* 5–1, *Rhodococcus sp.* MT11–4, *Sanguibacter keddieii* 11–6 and *Sphingomonas profundi* LMO-1 in the low-degree-of-homogeneity sample (sample III) were relatively low (<0.9). For *P. gessardii* 5–1, which should not have been present in sample Ib, the alignment results indicated gene detection (≤ 32%, false positive). This may be because *P. gessardii* 5–1 and *Pseudomonas veronii* 2–3 belong to the same genus, and some similar gene sequences might be detected incorrectly.

Additionally, metrics such as the mapping rate, read mapping status, and mismatch rate [46–48] were employed to rank the pipeline performances (Supplementary Table S5-S7). Based on the mapping rate, different combinations of trimmers and aligners showed varying performance, with Trimmomatic + BBMap yielding the best results (Fig. 3C). Similar results were obtained in the mapping status (Fig. 3D). Moreover, pipelines associated with BBMap exhibited no mismatches (Supplementary Fig. S1). This could be attributed to its incorporation of trimming during the alignment process.

### Evaluation of taxonomic profilers

**Figure 4:**
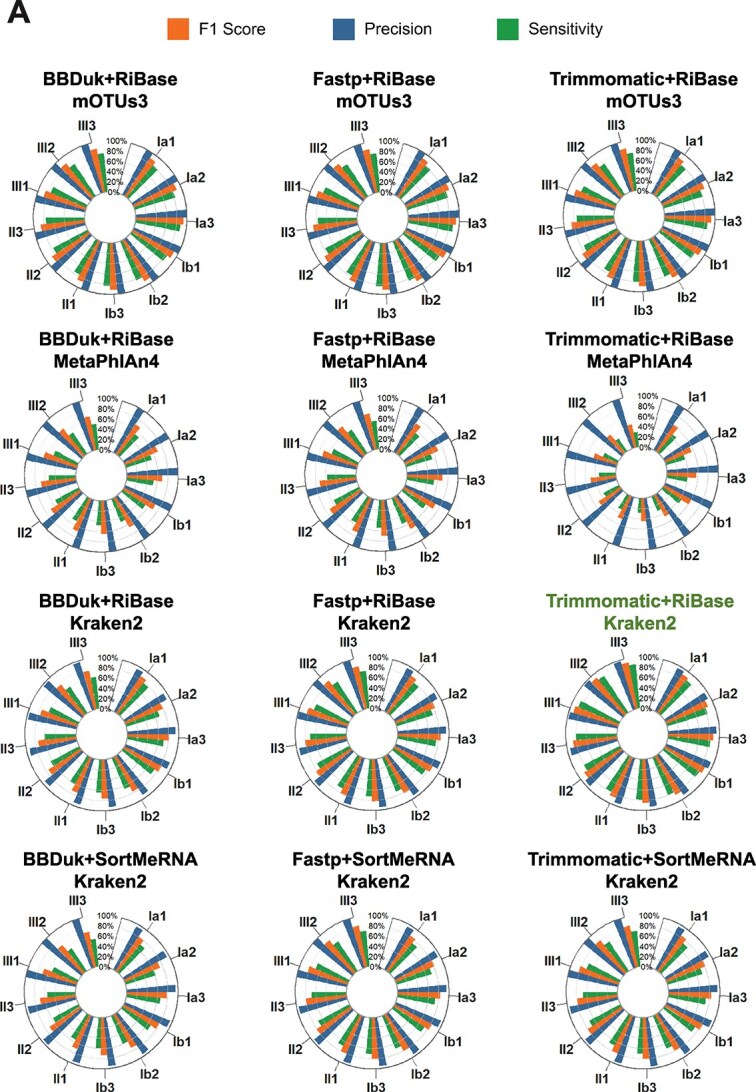
**Relative performance of taxonomic profilers with different error metrics (F1-scores, precision and sensitivity). A** Performance of MetaPhlAn4 and Kraken2 with different preprocessing pipelines.

Kraken2 (RRID:SCR_005484) is a widely used taxonomic profiler that identifies the taxonomic identities and relative abundances of microbial community genomes by comparing sequence reads against comprehensive metagenomic databases [49, 50]. MetaPhlAn4 (RRID: SCR_004915) and mOTUs3, in contrast, rely on reference databases containing only specific gene families for taxonomic annotation [50]. We performed taxonomic analyses using Kraken2, MetaPhlAn4, and mOTUs3 on pre-processed reads and evaluated their performance based on predefined species standards. The evaluation metrics included sensitivity, precision, and F1 score (Materials and Methods) [35], along with taxonomic rank-level assessments. Among these, the combination of Trimmomatic + RiBase + Kraken2 achieved superior performance, with all metrics scoring above 0.78 (Fig. 4).

During genus-level analysis of species detection rates, we found that mOTUs3 completely failed to identify *Sphingomonas* across all samples (detection rate: 0%). To investigate this limitation, we cross-referenced the Mock-Ref database with mOTUs3’s proprietary marker gene database to locate markers corresponding to the 10 studied strains (Materials and Methods). Our alignment analysis demonstrated that no marker genes in mOTUs3’s database could successfully map to the *Sphingomonas profundi* LMO-1 genome under stringent criteria (E-value <1e-5; ≥97% coverage of each marker gene’s full length). To examine potential taxonomic misassignment, we isolated all mOTUs3-annotated *Sphingomonas* markers and performed comparative alignment against the *Sphingomonas profundi* LMO-1 reference genome. The maximal sequence similarity observed (~80%) definitively confirmed the absence of LMO-1-specific markers in mOTUs3’s database. This case highlights potential limitations in the comprehensiveness and taxonomic precision of mOTUs3’s curated marker database for specific microbial species in this study.

Additionally, Kraken2 demonstrated satisfactory performance in distinguishing closely related species within the same genus, such as *P. gessardii* 5–1 and *P. veronii* 2–3. This observation aligns with prior evaluations of taxonomic classification tools [51]. However, a small number of false positives were observed, likely attributable to genetic similarities within the *Pseudomonas* genus.

### Transcript quantification

**Figure 5:**
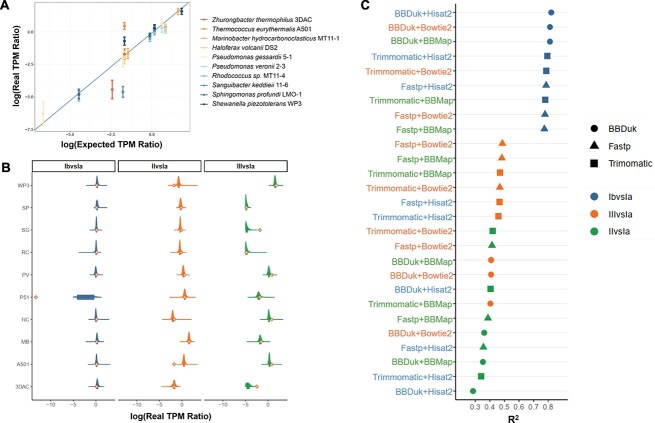
**Transcript quantification of MT-Enviro. A** Correlation analysis between theoretical values and actual TPM ratios obtained using the gold standard as the reference genome. The integrated pipeline used is Trimmomatic + RiBase + BBMap. The mean values of the logged TPM ratios among different strains and samples are shown with dots, and the error bars are expressed as the mean ± SD. **B** The semi-violin plot displays the log-transformed distribution of actual TPM ratios of genes across different samples, with dots indicating the results of log-transforming the theoretical TPM ratios. The column label Sample1vsSample2 indicates that for the unique genes of each strain on the y-axis, their TPM values in Sample1 are divided by their TPM values in Sample2 to calculate the actual TPM ratio in the real samples. The ratios for all unique genes of each strain are recorded and used to create the semi-violin plot. **C** The R^2^ values between the results obtained from different workflows and the theoretical ratios are displayed. The shapes of the points are used to distinguish between quality control software tools, while the colours of the points represent the fitting results of the ratios between different sample comparisons and the theoretical values. In the Y-axis labels, orange corresponds to Bowtie2, green corresponds to BBMap, and blue corresponds to HISAT2.

In practical scenarios of metatranscriptomic analysis, the precision of transcriptome quantification holds paramount importance. The accuracy of transcriptome quantification can be ascertained through a correlation analysis between theoretical values and quantification values such as TPM obtained from Mock-Ref [34]. The higher R^2^ value signifies the more accurate quantification. To ensure the precision of the quantitative comparisons, only TPM values for strain-specific nonredundant genes expressed across all samples were recorded. Since RNA-mixed samples derive from the RNA of individual strains combined in specific proportions, the gene TPM ratios from each strain across different samples should closely resemble the RNA contribution ratios from each strain. The linear formula for log2-transformed TPM ratios was fitted into Equation 1. (Fig. 5A).

Additionally, the examination of the distribution of actual TPM ratios relative to theoretical ones (Fig. 5B) reveals that the results closely align with theoretical expectations. Furthermore, by comparing the R^2^ values of the actual TPM ratios from different workflows to the theoretical ratios (Fig. 5C), we can evaluate the quantification accuracy of these workflows.


(1) \begin{align*}& {\log}_2\left(\mathrm{Real}\ \mathrm{TPM}\ \mathrm{Ratio}+0.0001\right)\notag\\&=k\bullet{\log}_2\left(\mathrm{Expected}\ \mathrm{TPM}\ \mathrm{Ratio}+0.0001\right)+b \end{align*}

### Overall performance of the MT-enviro strategy

**Figure 6:**
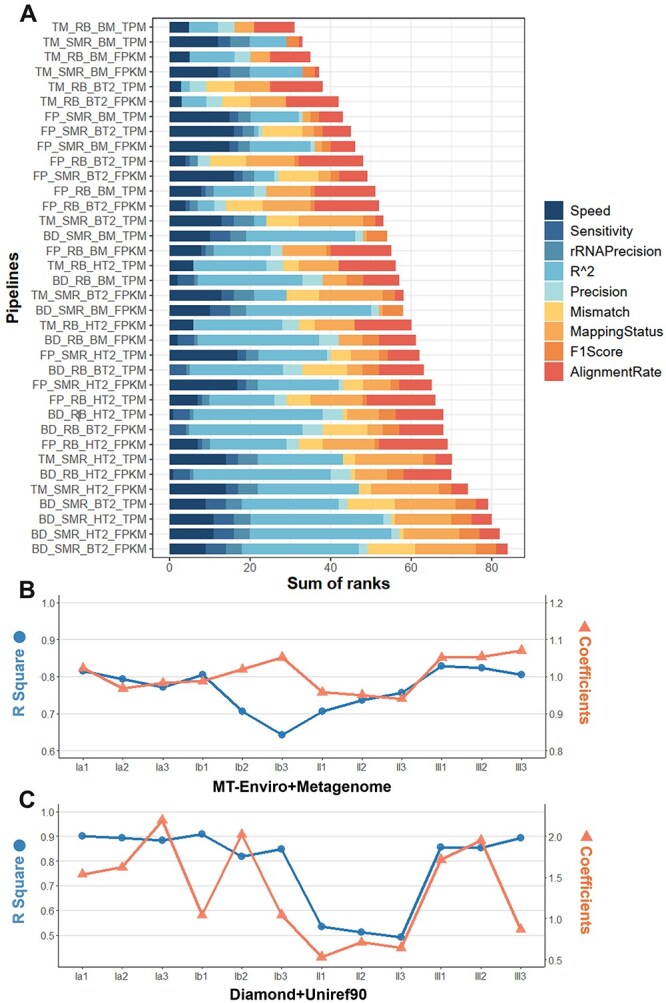
**Comparison of the overall performance. A** The chart displays the cumulative ranking for each pipeline based on multiple metrics including Speed, RNA Precision, Sensitivity, R^2, Precision, Mapping Status, Mismatch, Alignment Rate, and F1 Score. Pipelines are sorted by their summed ranks, with shorter bars indicating superior overall performance. Quality control, rRNA removal, comparison, and standardization methods are distinguished by underscores, and software names are abbreviated (TM-Trimomatic, FP-Fatsp, BD-BBDuk; RB-RiBase, SMR-SortMeRNA; BM-BBMap, BT2-Bowtie2, HT2-Hisat2). **B** Correlation analysis of TPM values obtained from Mock-Ref and metagenome. **C** Correlation analysis of TPM values obtained from Mock-Ref and Uniref90. For each sample, the R^2^ and correlation coefficients obtained by linear fitting of the TPM values of the same unique gene obtained from the standard genome are distinguished by different dot shapes.

The evaluation process encompassed a total of 36 combinations on 12 samples, involving three alignment tools (BBMap, Bowtie2 and HISAT2), two rRNA removal methods (RiBase and SortMeRNA), three quality control tools (Trimmomatic, BBDuk and fastp), and two reads standardization method (TPM and FPKM). To facilitate a straightforward assessment of the overall performance of each pipeline across various evaluation criteria, we ranked their performance under each evaluation criterion and selected the pipeline with the least ranking score as the best-performing one (Fig. 6A). For each evaluation criteria, rank 0 indicates the best performance (Supplementary Table S8).

It is evident that the pipeline comprising Trimmomatic + RiBase + BBMap + TPM exhibited the most robust overall performance. It not only ensures the accuracy and reliability of analytical outcomes but also enhances efficiency. In the subsequent research, this optimal pipeline has been adopted as the MT-Enviro strategy for the analysis of metatranscriptomic data. Moreover, it’s important to note that while this pipeline excels overall, it might not be the optimal choice for each specific step of the analysis, highlighting the necessity of a nuanced approach in tool selection.

### Real-world performance of MT-Enviro

In real-world metatranscriptomic analyses, a set of metagenomic data derived from the same experimental context is commonly used as a reference genome for reference-based analyses. For cases where metagenomic data are unavailable, some studies employ less stringent protein similarity search algorithms, such as DIAMOND [52], against databases like UniRef90 to obtain quantitative information [43].

To evaluate the real-world performance of the optimal pipeline, alignment results were assessed using three types of reference datasets: (i) Mock-Ref: Pooled reference genomes composed of 10 selected strains; (ii) Metagenome Reference: Assembled from metagenomic reads derived from the high-evenness cell-mixed sample (hIa) (Fig. 1D); (iii) UniProt Reference: UniRef90 database [38].

Reads were mapped against the Mock-Ref and Metagenome Reference using the MT-Enviro pipeline, while DIAMOND was used for alignment against UniRef90, simulating another quantitative analysis approach in real-world scenarios. Using the Mock-Ref quantitative results as a benchmark, the quantitative values obtained with the Metagenome Reference and UniRef90 were subjected to correlation analyses against the benchmark to evaluate whether alignment against these three references produced comparable results. Only strain-specific unique genes identified in all three references were selected as target genes.

The linear relationships between TPM values were modeled using Equations 2 and 3. The coefficients derived from the linear regression of each sample pair were highly significant (*P*-value <2.2e-16), and the quality of the fit was assessed using R^2^ and regression coefficient values (Fig. 6B and C). When comparing the quantitative values derived from Mock-Ref and UniRef90, the R^2^ values ranged from 0.491 to 0.908, with a mean of 0.783 across all 12 samples. The regression coefficients for these comparisons ranged from 0.533 to 2.196.

Similarly, the comparison between Mock-Ref and Metagenome-derived quantitative values revealed R^2^ values ranging from 0.635 to 0.914, with a mean of 0.766 across all 12 samples. The mean regression coefficient in this case did not significantly differ from 1 (Student’s t-test, *P*-value >0.05), with individual regression coefficients ranging from 0.940 to 1.071. Notably, the closer the regression coefficient is to 1, the greater the similarity between the two datasets. These findings confirm that the optimal pipeline performs robustly, yielding comparable results even when metagenomic data are used as a reference.


(2) \begin{equation*} \mathrm{TPM}\ \mathrm{from}\ \mathrm{metagenome}=k\bullet \left(\mathrm{TPM}\ \mathrm{from}\ \mathrm{Mock}\ \mathrm{Ref}\right)+b \end{equation*}

(3) \begin{equation*} \mathrm{TPM}\ \mathrm{from}\ \mathrm{Uniref}90=k\bullet \left(\mathrm{TPM}\ \mathrm{from}\ \mathrm{Mock}\ \mathrm{Ref}\right)+b \end{equation*}

## Discussion

The study of environmental microbial ecosystems requires large amounts of omics data to fully understand their properties and functions. However, the impact of the high heterogeneity and low annotation rates on the accuracy of analysis is still not well understood [4, 53–55]. To address this challenge, our study constructed 24 mock microbial communities with well-defined species compositions and proportions. A total of 659.05 Gb (Gbase) of genomic, metagenomic, and metatranscriptomic sequencing data were generated, which is 3 ~ 12 times larger than the sizes of currently available mock community datasets or *in silico* datasets [56, 57]. With these well-defined mock communities, we could directly assess the accuracies of taxonomic annotation and quantitative analysis via different pipelines. Furthermore, we are able to illustrate the independent effects of multiple factors, such as sample preparation, sequencing bias, pipeline efficiency, cellular type, genome size, community evenness and community complexity on metatranscriptomic analysis.

In this study, we proposed the MT-Enviro pipeline, designed to achieve high accuracy and efficiency. In the context of complex environment samples with varying evenness and different processing strategies, the combination of Trimmomatic + RiBase + Kraken2 + BBMap + TPM demonstrates stable and strong performance. Using mock communities, we confirmed that the MT-Enviro pipeline is suitable for different environmental samples. For example, the TPM values of the unique genes from the novel strain 3DAC did not significantly differ from the theoretical values (Fig. 5A and B). Additionally, the quality control software showed sensitivity to community evenness. In processing samples with low evenness, Trimmomatic performed better than BBDuk or fastp, although it required more time than BBDuk (Fig. 3A). This is consistent with previously published software evaluation findings [29, 58]. In the evaluation of taxonomy annotation, we found that the construction of marker gene databases is critical for profiling software that utilizes marker genes. It is essential to select accurate and representative marker genes as references for analysis, and to enhance the coverage of microbial species in the reference database. Otherwise, the accuracy of taxonomic profiling may be compromised. Our findings underscore that sample preparation techniques and community evenness can introduce variability in metatranscriptomic analyses, highlighting the need for improved accuracy in species annotation software. This suggests that relying solely on software-generated results might underestimate the true diversity and complexity of species within the dataset.

Additionally, we employed two distinct methods for preparing mock communities: RNA-mixed and cell-mixed methods. The significant differences between expected and actual RNA quantities in cell-mixed samples may be due to using OD_600_ values to infer biomass and RNA production, as OD measurements can lead to unreliable cell number estimations [59]. Another contributor is the variation in RNA extraction efficiency among different strains, which can vary up to 90-fold [60]. Natural environmental samples have much higher diversity than the mock communities and could suffer even stronger bias due to differences in RNA extraction efficiency. Moreover, transportation and repeated freeze–thaw cycles may also contribute to these discrepancies.

In our study, there are still some limitations worth noting. First, despite selecting ten microbes from diverse environments to construct simulated microbial communities, these communities may not fully represent real-world environmental samples. The uncertain annotation accuracy of organisms in real environments has long been a challenge for the accuracy of analysis tools. To ensure that our mock microbial communities are more reflective of real environmental samples while maintaining the dataset’s validity as a benchmark, we incorporated a mix of well-characterized strains (*Shewanella piezotolerans* WP3, *Haloferax volcanii* DS2), unpublished new isolates (*Rhodococcus* sp. MT11–4, *Marinobacter hydrocarbonoclasticus* MT11–1, *S. keddieii* 11–6), and, importantly, to address the inclusion of organisms without reference genomes, we specifically included *Zhurongbacter thermophilus* 3DAC. This strain was isolated and identified in our laboratory, and at the time of this study, its reference genome was unavailable in public databases. Phylogenomic studies of *Zhurongbacter thermophilus* 3DAC suggest that it may represent a novel thermophilic bacterial phylum, closely related to other thermophilic groups such as *Coprothermobacterota*, *Dictyoglomota*, *Caldisericota*, *Thermotogota* and *Thermodesulfobiota* [61]*.* This inclusion allowed us to evaluate the effectiveness of the analysis tools in handling gene alignments for organisms lacking reference genomes.

Another limitation is the fixed homogeneity of the metagenomic data. We posit that the uniform species distribution improved metagenome assembly completeness by ensuring consistent read coverage across low-abundance species. Consequently, contig fragmentation due to extremely low coverage was minimized. These findings are corroborated by the metagenome’s high coverage rate (83.90%) against the Mock-Ref database. However, it must be recognized that the uniform species proportions in hIa cannot fully replicate the impact of extreme abundance disparities on quantification accuracy in real-world contexts, potentially leading to overestimated detection sensitivity for low-abundance taxa. Furthermore, the even condition may inadequately reveal analytical algorithms’ sensitivity to abundance variations (e.g. inherent biases toward dominant species in certain tools), thereby constraining a comprehensive evaluation of methodological performance boundaries.

To bridge this gap while maintaining methodological rigor, we implemented a two-step strategy for quantitative validation. First, the RNA proportions of each microbe in different samples derived from Mock-Ref-based quantification were compared with the theoretical RNA proportions used to construct the simulated dataset, thereby validating the effectiveness of the Mock-Ref-based quantification results. Second, these results served as a reference to assess MT-Enviro’s performance under real-world simulations using both hIa-derived metagenome and DIAMOND+UniRef90 workflows. While this two-step strategy takes into account the influence of laboratory conditions and bioinformatics tools on result interpretation, incorporating spike-ins in future analyses could further improve the accuracy and reliability of our quantitative assessments [62]. Furthermore, benchmark testing was performed by keeping parameters as consistent as possible and close to their default settings, which might have influenced the outcomes. In our future work, the pipeline can also be considered in combination with the *in silico* simulation dataset to assess the analytical performance of the software. Given that our qualitative and quantitative analyses primarily utilized known genome compositions, further exploration of their potential in de novo analysis scenarios is warranted.

In summary, we have developed and validated an open-source metatranscriptomic pipeline, MT-Enviro. This flexible pipeline is adaptable to various environmental conditions, allowing for the integration of different tools. MT-Enviro is designed to analyse metatranscriptomic data from diverse environments, aiding in understanding the functional diversity and activity of microbes. Furthermore, both our mock-community datasets and the MT-Enviro pipeline can serve as valuable benchmarks for the metatranscriptomic analysis of environmental microbes.

## Supplementary Material

SUPPLEMENTARY_FIGURES_ycaf090

Supplementary_Tables_ycaf090

## Data Availability

The datasets generated and/or analysed during the current study are available in the National Omics Data Encyclopedia repository (NODE) under accession number project OEP00003879.

## Code availability

The MT-Enviro source codes and the integrated pipeline are available in GitHub (https://github.com/Li-Lab-SJTU/MT-Enviro).
