# Supplementary material for: Benchmarking and optimizing qualitative and quantitative pipelines in environmental metatranscriptomics using mixture controlling experiments
Source: ISME Commun. 2025 May 29;5(1):ycaf090. doi: 10.1093/ismeco/ycaf090 (PMC12202999; doi:10.1093/ismeco/ycaf090)
Supplement: SUPPLEMENTARY_FIGURES_ycaf090 [file supplementary_figures_ycaf090.pdf]

SUPPLEMENTARY FIGURES

Benchmarking and optimizing qualitative and quantitative pipelines in environmental metatranscriptomics using mixture controlling experiments

Li et al.

Includes:

Supplementary Fig. 1-4

Supplementary Fig. 1

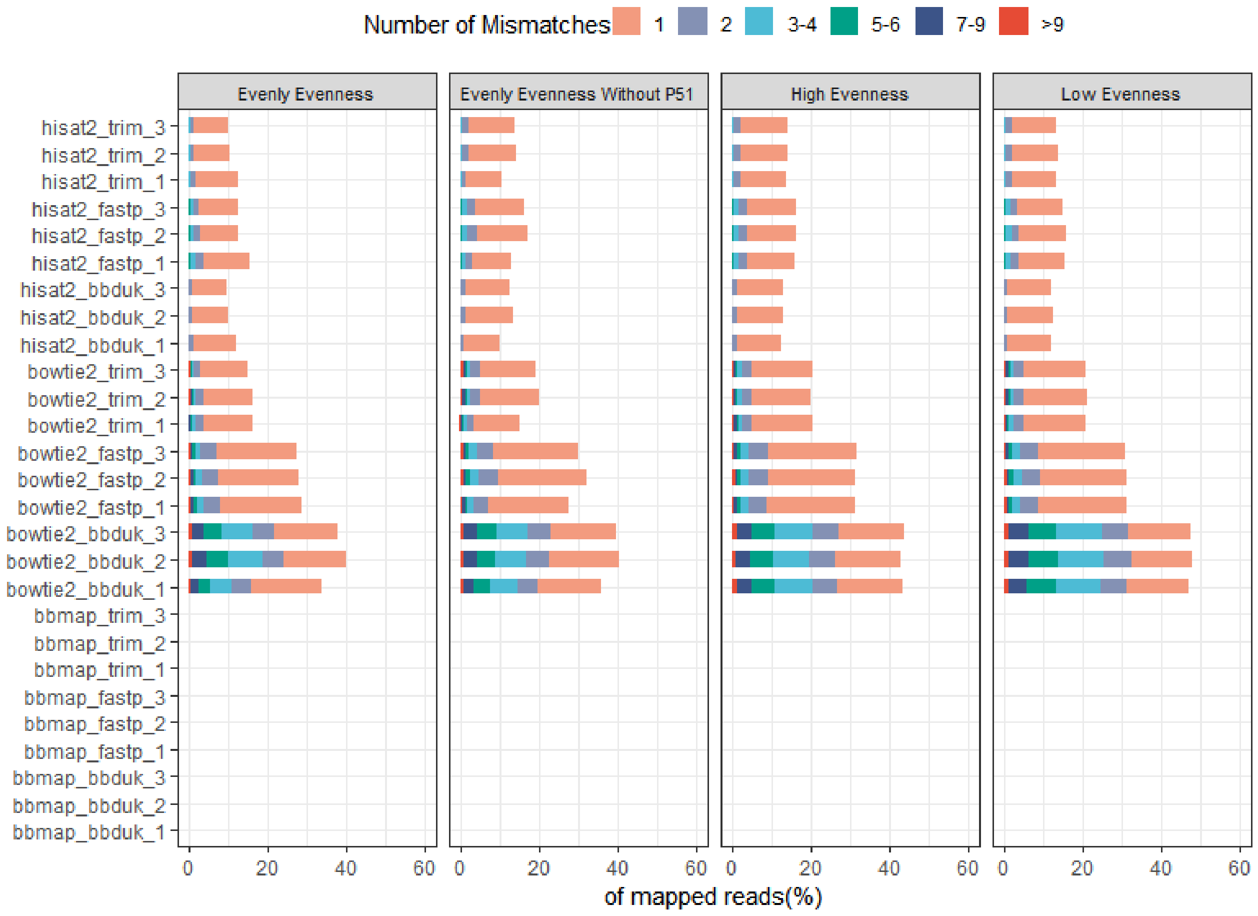

Supplementary Fig. 1. Distribution of mismatches across nine reference-based alignment pipelines. The y-axis represents the analysis results of triplicate samples from each pipeline, while the x-axis shows the proportion of reads with mismatches during mapping. Reads with different numbers of mismatches are distinguished by different colors.

Supplementary Fig. 2

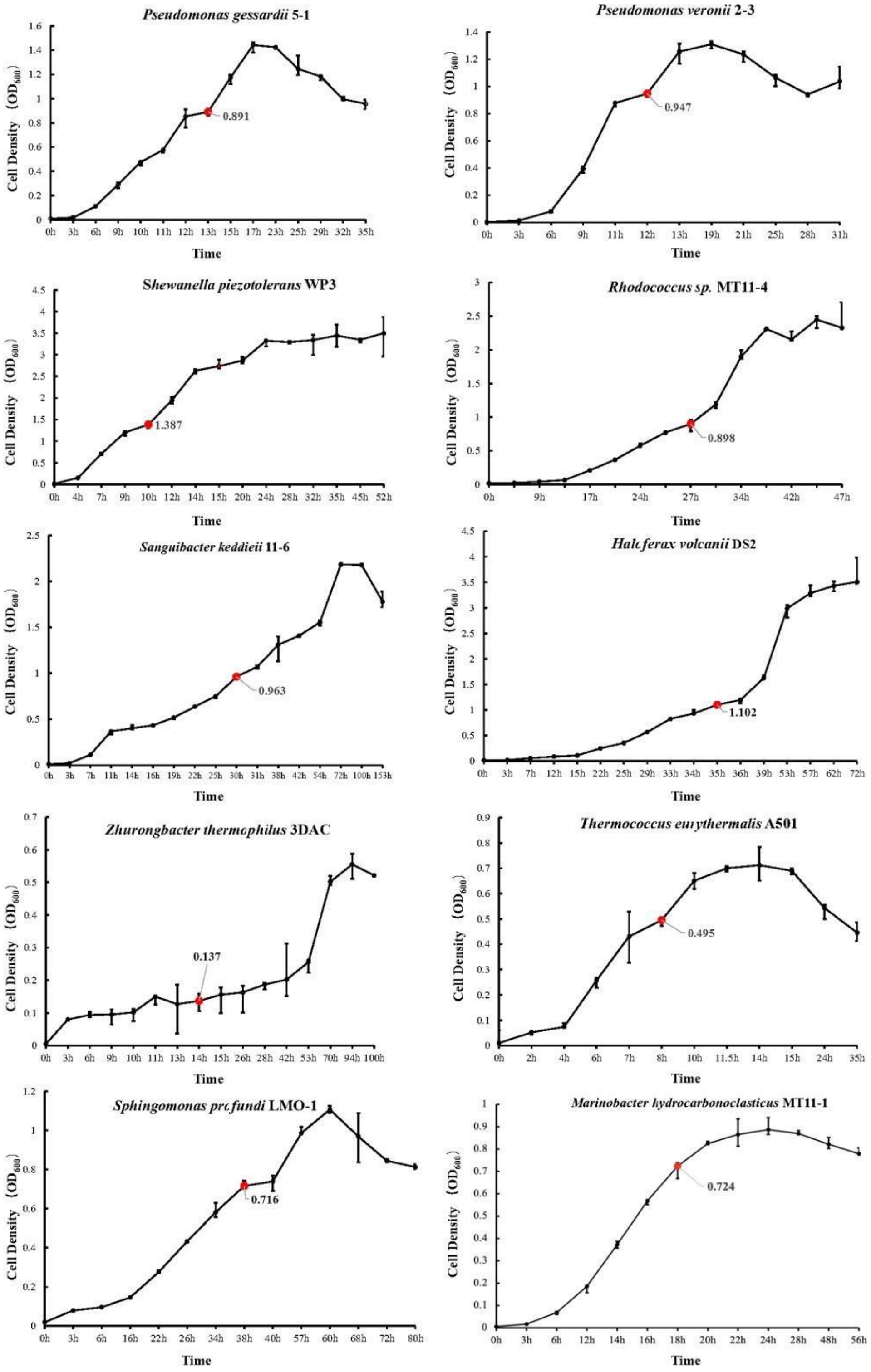

Supplementary Fig. 2. Growth curves and sample collection points of each strain. Sampling points for RNA extraction are indicated by red dots. Data represents mean and standard deviation obtained from three independent experiments.

Supplementary Fig. 3

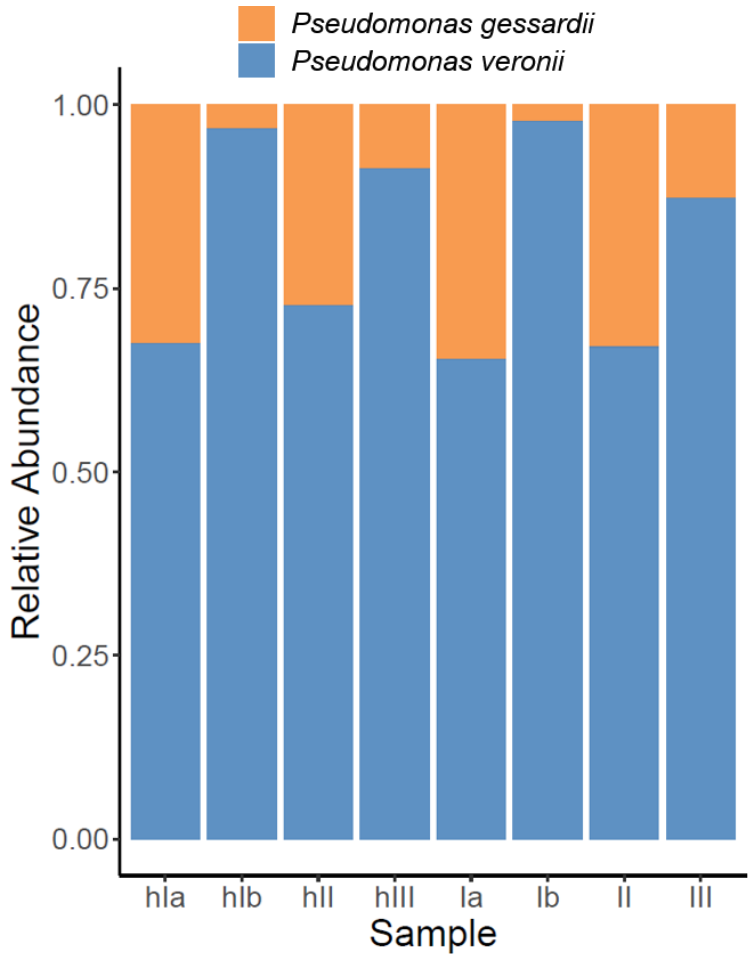

Supplementary Fig. 3. Bar chart of species annotation results from the same genus. The relative abundances of *Pseudomonas gessardii* 5-1 (P51) and *Pseudomonas veronii* 2-3 (PV) in different samples are shown at the species level.

Supplementary Fig. 4

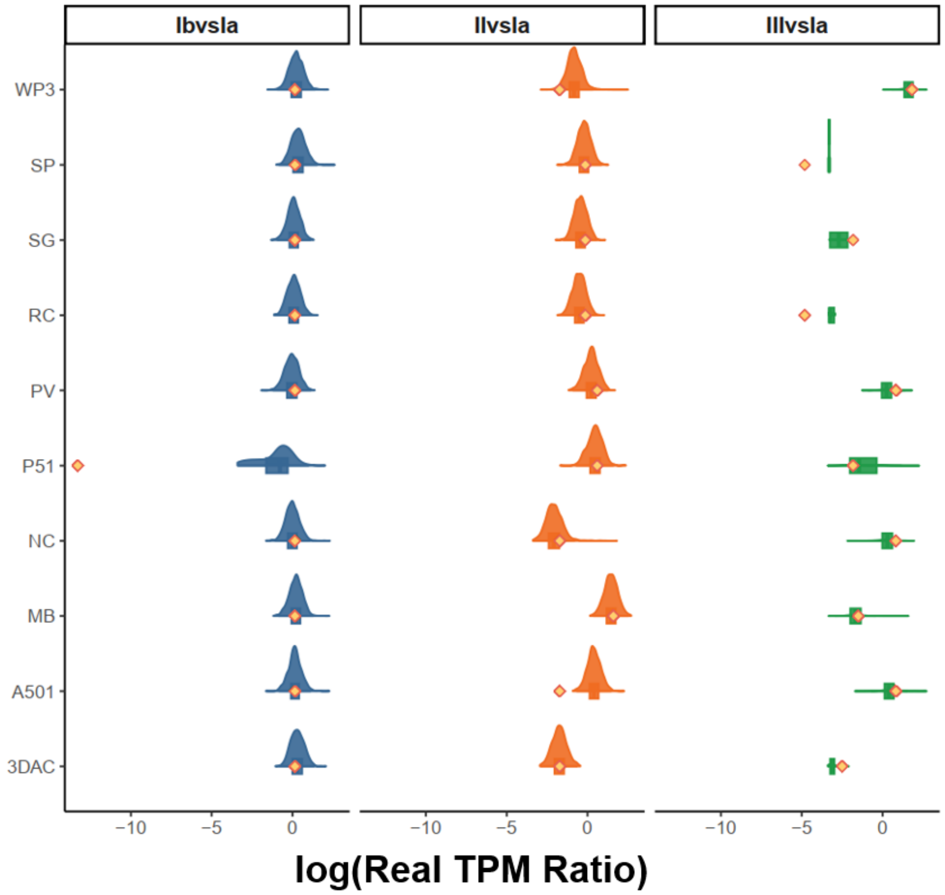

Supplementary Fig. 4. TPM Ratio from Uniref90-based quantification analysis. The semi-violin plot displays the log-transformed distribution of Uniref90-based TPM ratios of genes across different samples, with dots indicating the results of log-transforming the theoretical TPM ratios. The column label Sample1vsSample2 indicates that for the unique genes of each strain on the y-axis, their TPM values in Sample1 are divided by their TPM values in Sample2 to calculate the actual TPM ratio in the real samples.
